# Adolescent empathy predicts reduced neural responses to social rejection in adulthood

**DOI:** 10.1017/S095457942610131X

**Published:** 2026-03-04

**Authors:** Jingrun Lin, Jacob Moore, Nathan Field, Jessica Stern, Joseph Allen, James Coan

**Affiliations:** 1 Psychology, University of Virginia, USA; 2 Psychological Science, Pomona College, USA

**Keywords:** adolescence, Cyberball, empathy, fMRI, rejection sensitivity

## Abstract

**Objective:**

Adolescence is a sensitive period for social and neural development. Empathic growth during adolescence has been linked to improved prosocial behavior in adulthood. This study examined how adolescent empathy relates to adulthood neural responses to rejection.

**Method:**

Participants (*N* = 77; 42 females, 52% White) were drawn from a demographically diverse community sample and assessed annually from ages 13 to 21. Each year, participants’ empathic support provision toward a close friend was evaluated during an observationally coded support task. At approximately age 24, participants completed the Cyberball social exclusion paradigm while undergoing fucntional magnetic resonance imaging (fMRI).

**Results:**

Whole-brain exploratory analyses revealed that greater empathic support provision during adolescence was associated with reduced activation in the subgenual anterior cingulate cortex (sACC) during social exclusion in early adulthood (Cohen’s *d* = 0.12), suggesting a contribution of empathy provision to rejection-related neural responses later in life. The effect was not driven by felt distress during social exclusion, indicating that adolescent empathic support provision is potentially associated with neural responses to social exclusion independent of subjective distress.

**Conclusion:**

These findings underscore the long-term links of empathy to adult social processes and may inform interventions aimed at enhancing interpersonal functioning and resilience.

Rejection sensitivity refers to the tendency to anxiously expect, readily perceive, and intensely react to social rejection (Downey & Feldman, 1996). Although awareness of the potential for social disapproval can motivate repair of social connections, hypersensitivity to social rejection is an interpersonal vulnerability factor contributing to difficulties forming and maintaining satisfying social relationships (Downey et al., 1998; Marston et al., 2010). Additionally, rejection sensitivity has been linked to various clinical health outcomes, including depression, anxiety, loneliness, borderline personality disorder, and body dysmorphic disorder (Gao et al., 2017). Emerging research further suggests that rejection sensitivity is associated with distinct neural activation patterns, particularly in regions involved in affective processing and cognitive control (Eisenberger et al., 2003). But why do some individuals feel so intensely hurt, or even threatened, by subtle cues of disconnection?

Empirical research has identified several developmental factors that predict rejection sensitivity. For instance, childhood maltreatment, including physical and emotional abuse (Gao et al., 2024), early problematic relationships with parents and peers (Araiza et al., 2020), and exposure to family violence (Feldman & Downey, 1994), have all been linked to elevated rejection sensitivity in later life. While most research has examined how experiences of receiving care shape rejection sensitivity, the present study focuses on adolescents’ provision of empathic support, a complementary process reflecting the caregiving system that may confer protection against social threat. The present study contributes to this line of research by tracking adolescents’ support processes in close friendships from ages 13 to 21 to examine how peer-related social interactions, particularly empathic support, are linked to rejection-related neural responses in early adulthood as assessed through fMRI.

## Social rejection & the brain

One method of measuring rejection sensitivity at the neural level is through the Cyberball paradigm – a computerized ball-tossing game adapted for an fMRI design. Eisenberger and colleagues (2003) published the first neuroimaging study of social rejection using the Cyberball paradigm, demonstrating increased activation in the dorsal anterior cingulate cortex (dACC) and ventral prefrontal cortex during exclusion relative to inclusion. Notably, ventral prefrontal cortex activity was negatively correlated with dACC activation, suggesting that prefrontal regions may play a regulatory role in modulating distress associated with social exclusion. Subsequent Cyberball studies have identified a broader network of regions engaged during exclusion, including the ventral anterior cingulate cortex, anterior insula, and lateral prefrontal regions, highlighting the distributed nature of neural responses to social rejection (e.g., Masten et al., 2009; Onoda et al., 2009).

Building on this work, a growing literature has emphasized functional heterogeneity within the anterior cingulate cortex and its interactions with prefrontal and limbic regions during rejection-related experiences. Meta-analytic evidence indicates that multiple subdivisions of the ACC, including dorsal, pregenual, and subgenual regions, are reliably engaged during social rejection and self-reported distress. In particular, while dorsal ACC corresponds to the processing of cognitive conflict, ventral ACC corresponds to specific social and emotional evaluation (Somerville et al., 2006). At the same time, the functional significance of ACC activation may also vary as a function of developmental stage, task demands, and broader contextual factors. Developmental neuroimaging studies indicate that children and adolescents often exhibit heightened ACC responsivity to rejection relative to adults, in line with greater socioemotional sensitivity earlier in development (Bolling et al., 2011; Rotge et al., 2015). Likewise, differences in task structure such as the length, design, and expectation of rejection may also bias differential ACC engagement (Rotge et al., 2015). Together, these findings suggest that ACC responses to social rejection are dynamic and context-dependent, reflecting shifting contributions across developmental and experimental contexts.

In addition to ACC involvement, prefrontal regions have been consistently implicated in the regulation of affective responses to social exclusion. For example, the ventral and lateral prefrontal cortices, including right ventrolateral prefrontal cortex, have been associated with downregulation of limbic and salience-related responses during exclusion (Eisenberger et al., 2003). Moreover, prefrontal engagement during exclusion appears to vary with individual differences in regulatory capacity and emotional context; for instance, greater right ventrolateral prefrontal cortex activation has been linked to more effective self-regulation in daily life when felt rejection is low (Chester & DeWall, 2014). Together, these findings underscore that neural responses to social exclusion reflect coordinated activity across distributed affective, cognitive, and regulatory systems. Given overlapping circuits implicated in affective psychopathology (i.e., depression), individual differences in neural responses to social rejection may reflect broader affective vulnerability and individual resilience (Drevets et al., 1997; Hamani et al., 2011).

## Peer experience & neural sensitivity to rejection across adolescence

Adolescence represents a sensitive developmental period characterized by increasing independence from caregivers and heightened reliance on peer relationships (Jaworska & MacQueen, 2015). During this time, peer interactions serve as a critical context for acquiring social competencies that support adult interpersonal functioning (Allen et al., 2020). As such, individual differences in how strongly the brain responds to social rejection in adulthood may have developmental roots in these adolescent peer experiences, which inform individuals how to anticipate, interpret, and cope with rejection in social relationships later in life.

This developmental sensitivity is mirrored at the neural level. Adolescence is marked by substantial structural and functional brain reorganization, including changes in fronto-subcortical connectivity that support increasingly complex social cognition while rendering the brain particularly responsive to social input (Paus et al., 2008; Pfefferbaum et al., 1994). Empirical evidence supports this link: peer preference, exposure to chronic rejection, and repeated victimization were associated with heightened activation in the lateral prefrontal, supramarginal, striatal, and insular gyrus activity in response to social exclusion during the Cyberball task (Asscheman et al., 2020; Kellij et al., 2024; Will et al., 2016). Such findings suggest that adverse peer experiences may sensitize individuals to future rejection, whereas positive peer interactions may offer some protection. Peer experiences, especially those involving exclusion or acceptance, may therefore calibrate neural and affective systems that detect and regulate social threat.

## Empathy as a bridge between peer experiences and rejection sensitivity

While adverse peer experiences sensitize adolescents to social threat, empathy may function as a key mechanism that moderates these effects. Empathy develops substantially during adolescence and supports the formation of supportive peer relationships and social competence (Allemand et al., 2015; Costello et al., 2023; Field et al., 2025) that reduce vulnerability to social threat (Portt et al., 2020). Neuroimaging evidence further supports a close link between empathy and rejection-related neural processing. For example, Beyer et al. (2014) found that individuals who experienced social exclusion showed heightened reactivity in cognitive mentalizing and the mirror neuron system (bilateral superior, middle and inferior temporal gyrus, bilateral precuneus, right precentral gyrus) when viewing emotional scenes; furthermore, neur reactivity was positively correlated with aggressive behavior in a subsequent experimental paradigm. Similarly, when adolescents and adults observe others being socially excluded, activation in the mentalizing regions (medial and dorsomedial prefrontal cortex, temporoparietal junction) and social-pain regions (anterior insula, dACC) predicts empathic concern and subsequent prosocial behavior toward excluded peers (Masten et al., 2011; Meyer et al., 2015), linking key components of empathy to neural activities in response to social exclusion.

Importantly, empathy is a multifaceted construct encompassing both cognitive and affective components that may have distinct implications for social functioning and emotion regulation. Cognitive empathy – the capacity to understand and accurately infer others’ mental states, intentions, and perspectives – may facilitate more effective regulation in socially evaluative contexts, thereby reducing misperceptions that could heighten rejection sensitivity. Supporting this distinction, longitudinal evidence indicates that higher cognitive empathy predicts lower rejection sensitivity during adolescence, whereas greater personal distress predicts heightened sensitivity (Tan et al., 2023). Complementary evidence suggests that social exclusion attenuates later-stage empathic processing of others’ pain, downregulating cognitive components of empathy over time but does not affect empathic responses during the early emotional sharing stage (Fan et al., 2021). Additionally, Steinhoff and Keller (2020) found that childhood and adolescent sociomoral sensitivity (a correlate of cognitive empathy) protects adolescents from peer rejection and predicts early adulthood friendship intimacy, suggesting that sociomoral sensitivity developed early in life plays a critical role in shaping future relationships. Together, these findings position empathy, and especially its cognitive component as a critical neural and behavioral bridge linking adolescent peer experiences to later sensitivity to social exclusion.

Finally, empathy is inherently reciprocal. Adolescents’ provision of empathic support and the support they receive from peers may exert distinct yet interacting influences on neural responses to rejection. Prior findings showing both attenuated and heightened activation in regions including the dorsal anterior cingulate and anterior insula as a function of peer involvement (Masten et al., 2009, 2012). These mixed findings highlight the need to consider both sides of the empathic exchange–empathic support provision from teens and friends–to disentangle these processes and understand their unique contributions.

## Current design and hypotheses

The present study used a longitudinal design to test the prospective link between empathic support for peers during adolescence and neural sensitivity to rejection in early adulthood. We focused on teens’ empathic support for their close friends through an observationally coded supportive behavior task assessed annually from ages 13 to 21. Around age 24, we used the Cyberball paradigm to test for neural correlates of social rejection. As previous research showed that peer-related experiences such as peer preference (Susanne Asscheman et al., 2020) and time spent with peers (Masten et al., 2009, 2012) are linked to neural responses to social rejection during Cyberball, we aimed to extend this work by examining how both teens’ and friends’ empathic support provision may be linked to neural sensitivity to social rejection in early adulthood. Given substantial heterogeneity in both the neural systems implicated in social exclusion and the direction of empathy-related neural effects reported in prior research, hypotheses were exploratory rather than region-specific or directionally constrained.

Accordingly, we conducted whole-brain analyses to examine whether individual differences in adolescent empathic support provision were associated with neural responses to social exclusion relative to inclusion in adulthood. This approach allowed us to identify potentially distributed neural patterns, including regions implicated in mentalizing, affective processing, and regulatory control, as well as regions less commonly emphasized in the empathy or social exclusion literatures.

Additionally, to examine the dyadic context of empathic support, we conducted follow-up ROI analyses based on clusters identified in the exploratory whole-brain analyses. In these models, both adolescents’ own empathic support provision and their friends’ empathic support provision were included simultaneously while accounting for their inherent covariance. These analyses explored whether adolescents’ empathic support provision uniquely explains variance in neural responses to exclusion relative to inclusion in early adulthood, after accounting for empathic support received from friends.

Finally, we explored whether subjective distress during social exclusion, indexed by self-reported Need–Threat Scale (NTS) scores during the Cyberball task, mediated the association between adolescent empathic support provision and exclusion-related neural activation in regions identified in the ROI analyses. These analyses were designed to probe whether felt distress may explain observed associations between adolescent empathy and neural responses to exclusion. All analyses were adjusted for gender and baseline family income, given previous research demonstrating gender and socioeconomic status-related differences in empathy and supportive behaviors (Loeb et al., 2021; Van der Graaff et al., 2014).

**Preregistration note.** The preregistered analysis plan (https://osf.io/zx5vg/) specified a directional association in which higher adolescent empathic support was predicted to relate to reduced activation in exclusion-related neural regions. However, in response to mixed findings in the prior literature regarding the direction of empathy-related neural effects, the present manuscript treats all neural associations as exploratory and directionally unspecified. This reflects a change in inferential framing rather than a change to the analytic strategy or variables examined.

## Method

### Participants

The present report draws from a longitudinal, multi-reporter study of adolescent social development in familial and peer contexts. A sample of 184 seventh- and eighth-grade students, 99 of whom identified as female and 85 as male, were recruited from a public middle school drawing from suburban and urban populations in the Southeastern United States. Participants were assessed annually beginning in 1998, when the participants were age 13. The sample was racially/ethnically and socioeconomically diverse: 107 adolescents (58%) identified as white, 53 (29%) as African American, 15 (8%) as of mixed race or ethnicity, and 9 (5%) as being from other identity groups. Adolescents’ parents reported an annual median family income in the $40,000–$59,999 range at the time of initial assessment.

Drawing from this longitudinal sample, this study utilizes observational data collected annually from age 13 to 19 and again at age 21. Each year, target participants nominated their closest friend to participate with them in the study. Close friends were characterized as “people you know well, spend time with, and whom you talk to about things that happen in your life.” Participating close friends were same-gender peers close in age to the target participants at the time of data collection (on average, ages differed by less than a month between target adolescents and their close friends). Additionally, this study utilizes fMRI data drawn from a neuroimaging task completed by target participants around age 24.

For the present investigation, participants from the larger sample were included if they completed at least one of the observational assessments and the neuroimaging task. According to safety standards for fMRI practice, participants were excluded if they were pregnant, claustrophobic, or if they had ferromagnetic items in their body. Of those scanned, two participants were excluded due to prior knowledge of the fMRI task used in the study, and eight participants were dropped due to technical errors during data collection or abnormal neural activation resulting from poor image registration or structural abnormality. A total of 77 participants (42 females, 35 males) made up the final sample. Forty participants identified as white, 26 as African American, 6 as of mixed race or ethnicity, 2 as Hispanic/Latinx, 1 as Asian/Asian American, and 2 as being from other identity groups. The subsample included in the analyses did not differ significantly from the excluded sample in terms of empathy scores (age 13: *p* = .539, age 14: *p* = .939, age 15: *p* = .408, age 16: *p* = .172, age 17: *p* = .703, age 18: *p* = .440, age 19: *p* = .832) except for the last timepoint due to smaller sample size (age 21: *p* = .044). The subsample also did not differ from the excluded sample in terms of gender (*p* = .865). Socioeconomic status approached, but did not reach, significance (*p* = .050), indicating only marginal differences between groups. Empathic support provision scores were approximately normally distributed across all waves, with skewness values ranging from –0.49 to 0.33 and kurtosis values ranging from –0.46 to 0.16, indicating no substantial deviations from normality or evidence of outliers. All procedures were approved by the Institutional Review Board at the University of Virginia. Prior to age 18, adolescents provided informed assent and their parents provided informed consent before participating. Once they reached age 18, participants began providing informed consent. The data used in these analyses were derived from a larger longitudinal study, but the analysis plan was preregistered. The preregistration and analysis code are available online: https://osf.io/zx5vg/.

### Measures

#### Support processes (ages 13–19, 21)

Target participants and their nominated closest friend completed the Supportive Behavior Task (Allen et al., 2001), a 6-minute dyadic interaction designed to measure the quality of participants’ empathic support provision. Each dyad completed two interactions: in the first, the target participant asked their close friend to present a “problem they were having that they could use some advice or support about,” and in the second, the friend asked the target participant to present a problem. Empathic support provisions were coded separately for each task using the Supportive Behavior Task Coding System (Allen et al., 2001), allowing us to quantify empathic support provided by the target participant toward their friend and by the friend toward the target participant. All interactions were coded by two independent raters, and scores were averaged across coders to enhance reliability. To minimize potential bias, different coders were assigned to the target teen’s and the friend’s supportive behavior tasks. Notably, correlations between teen and friend support provision were modest and varied widely across waves (*r* = .51 to *r* = .01). Both target participants’ empathic support provision and friends’ support provision are measures well-validated in previous research (e.g., Stern et al., 2021, 2024).

*Target participants’ empathic support provision*. reflects the target teen’s emotional support provision, instrumental support provision, interpretation of the close friend’s problem, and emotional engagement. *Emotional support provision* reflects the extent to which participants attempt to support close friends by expressing understanding, labeling emotions, eliciting further emotional expression, or indicating emotional availability. *Instrumental support provision* reflects participants’ awareness of a friend’s problem and their efforts to help identify solutions, including recognizing the problem, offering solution-oriented ideas, and maintaining focus on resolving the issue. *Interpretation of the close friend’s problem* captures the extent to which the participant accurately recognizes and understands the issue raised. *Emotional engagement* reflects the degree to which the participant appears connected and emotionally engaged with the friend, independent of support content, as indicated by active listening behaviors such as following up, allowing space to speak, and asking relevant questions. Scores range from 0 to 4 (0 = little support/engagement/thoughtful interpretation; 4 = high levels of all of these factors, overtly demonstrating real connection, interest, and depth). ICCs for the target teen’s empathic support provision score at each time point ranged from .67 to .84, in the “good” to “excellent” range for this statistic (Cicchetti & Sparrow, 1981). Of note, our coding system reflects components of cognitive empathy in comparison to affective aspects of empathy and emotional resonance.

*Close friends’ empathic support provision* was observed using the Supportive Behavior Task (Allen et al., 2001) where the target participant *received* support from their close friend in response to a self-selected problem, and the close friend’s supportive behaviors were later coded using the Supportive Behavior Task Coding System (Allen et al., 2001). At each of the eight time points, teens’ close friends’ empathic support provision reflected the friends’ provision of *emotional* and *instrumental support, emotional engagement, and interpretation of the teen’s problem. Friends’ empathic support provision* was coded using the same scales described above (scores ranging from 0 to 4). ICCs for the support provision score at each time point ranged from .73 to .81, in the “good” to “excellent” range for this statistic (Cicchetti & Sparrow, 1981).

#### Cyberball fMRI paradigm (around age 24)

Target participants returned to complete a widely-used virtual task known as Cyberball (Williams et al., 2000) when they were around age 24 (*M*_age_ = 23.70; *SD* = .98). The Cyberball task involves participants playing a virtual ball-tossing game while undergoing functional MRI scans. Participants were told that they would be playing the game in real time with other participants undergoing the same study in other facilities. However, the two other “players” were not real: all of their movements were pre-programed. After providing informed consent, all participants were asked to write a short autobiography for the other players to read, and they were provided with autobiographies attributed to the two hypothetical players in turn.

Participants then entered the fMRI scanner and completed two rounds of the ball-tossing game, each lasting 2.5 minutes. A functional fMRI scan was completed during each round. Participants controlled an avatar represented by a hand in the lower middle of the screen, and two full-bodied cartoon avatars in the upper left and upper right corners of the screen represented the other “players.” Both rounds of the Cyberball game began with the avatar in the upper left tossing the ball either to the target participant or the other avatar. Tosses from both computer avatars were jittered between 0.2 to 2 seconds to maintain the impression of real human players. In the first round of the game, each pre-programed avatar tossed the ball to the target participant and the other pre-programed avatar equally, but in the second round, the participant only received the ball 10 times at the beginning of the game and then was ignored for the remainder of the session (50–60 seconds) and forced to watch the other “players” toss the ball between themselves. Previous research shows that this task is a reliable way of inducing feelings of social rejection (Eisenberger et al., 2003; Williams et al., 2000).

#### Need threat scale

Participants completed the 12-item version of the Need Threat Scale (Jamieson et al., 2010) following Cyberball, based on previous research that utilized the scale to assess for felt distress during the paradigm (Eisenberger et al., 2003). The scale included 12 items assessing for states of belonginess (e.g., “I felt rejected”), self-esteem (e.g., “I felt good about myself”), control (e.g., “I felt powerful”), and meaningfulness (e.g., “I had a feeling that my presence during the game was important”) during the Cyberball paradigm. Participants endorsed items on a 5-point Likert scale ranging from “not at all” to “very much so.” The average score represented the degree of felt distress (*M* = 2.43, *SD* = 0.70, range: 1–4.25, Cronbach’s alpha = .85).

#### Image acquisition and data analysis

Functional images were acquired using Siemen’s 3 tesla MAGNETOM Trio high-speed magnetic resonance imaging device. Target participants viewed Cyberball using the fMRI’s 12-channel head-coil with an integrated mirror. Prior to obtaining functional images, 176 high-resolution T1-magnetization-prepared rapid-acquisition gradient echo images were acquired to determine the localization of function (1-mm slices, TR = 1900 ms, TE = 2.53 ms, flip angle = 9°, FOV = 250 mm, voxel size 1 x 1 x 1 mm). After the anatomical scan, seventy-five functional T2*-weighted echo planar images sensitive to blood-oxygen-level-dependent contrasts were collected for each round of Cyberball (slice gap = 1-mm; slice thickness = 3.5 mm; TR = 2000 ms; TE = 40 ms; flip angle = 90°; FOV = 192 mm; image matrix = 64 mm x 64 mm; voxel size = 3 mm x 3 mm x 4.27 mm). These T2*-weighted echo planar images were collected in volumes of twenty-eight 3.5-mm slices, each with a 1-mm slice gap, covering the whole brain. Imaging data were preprocessed and analyzed using FMRIB Software Library (FSL) software (Version 5.98; www.fmrib.ox.ac.uk/fsl; 37). All fMRI images were skull-stripped using the BET brain extraction tool to eliminate non-brain material voxels (Smith, 2002). Additionally, the functional images were corrected for motion using FMRIB’s Linear Image Registration Tool and intra-modal correction algorithm, with slice scan time correction and a high-pass filtering cutoff point of 100 s to remove irrelevant signals (MCFLIRT; Jenkinson et al., 2002). Finally, a 5-mm full width at half-minimum Gaussian kernel was used for smoothing. (MCFLIRT; Jenkinson et al., 2002).

Functional images were registered to the Montreal Neurological Institute (MNI) standard space using FLIRT (Jenkinson et al., 2002). First- and second-level analyses were conducted using the FMRI Expert Analysis Tool (FEAT; Version 6.00) in FSL. For the Cyberball task, as in previous imaging studies, each round of Cyberball was modeled as a run containing both inclusion and exclusion blocks and individual responses to rejection were modeled using the second run of the paradigm only. Inclusion was modeled using the first inclusive 10 throws of the second run, and exclusion was modeled using the remaining exclusion throws. For each participant, an exclusion > inclusion contrast was computed at the first-level to estimate neural responses to social rejection.

At the second level, individual exclusion > inclusion contrast maps were entered into whole-brain analyses to examine the associations between teens’ and friends’ empathic support provision and neural sensitivity to social rejection. Whole-brain cluster correction was applied using a threshold of *z* = 2.3 and *p* = .05 (cluster corrected). Clusters were defined by structural probability maps provided in FSL and EBRAINS, along with continuity of functional activation to delineate boundaries of significant clusters.

In addition to the exploratory whole-brain analyses, we planned to conduct follow-up ROI analyses based on significant clusters identified from these whole-brain results. This two-step approach allowed us to first explore where empathic support provision across adolescence were most strongly associated with neural responses to social rejection in the brain, and then to confirm these effects within functionally or anatomically defined ROIs. Specifically, for each significant cluster emerging from the whole-brain analyses, we generated corresponding ROI masks using either functional meta-analytic maps from neurosynth.org or anatomical masks from the Harvard–Oxford Cortical and Subcortical Structural Atlases. All ROI masks were binarized using FSL’s cluster command, and the mean *Z*-statistics from the first-level exclusion > inclusion contrast were extracted within these regions for follow-up analyses. This analytic framework enabled us to complement exploratory whole-brain findings with confirmatory ROI analyses that allowed us to further explore the dyadic relation between teens’ and friends’ empathic support provision while accounting for their covariance.

#### Covariates

All analyses were adjusted for baseline family income (calculated as a percentage of the federal poverty line, which takes into account household size) and participant gender (male = 1, female = 2), based on previous research suggesting that empathy and supportive behaviors may vary by gender and socioeconomic status (Loeb et al., 2021; Van der Graaff et al., 2014). Consistent with the preregistered analysis plan, primary results are first reported from unadjusted models. All numerical data for continuous variables were mean centered prior to analysis.

## Results

### Preliminary analyses

To index teens’ and friends’ empathic support provision across adolescence, we computed composite scores by averaging each individual’s ratings across waves and used these scores in all subsequent neural analyses. Conceptually, this approach reflects our aim to capture a cumulative, trait-like indicator of empathic support provision rather than discrete, time-specific behaviors, which vary naturally as different social partners participate across years. Empirically, the composite score for teens’ empathic support provision demonstrated acceptable internal consistency across ages (Cronbach’s *α* = .73; Tavakol & Dennick, 2011). To further evaluate the robustness of this aggregate approach, we conducted supplementary analyses in response to negative correlations between adolescents’ empathic support provision at the final assessment wave and earlier timepoints. Specifically, we repeated analyses excluding the final wave and restricting the sample to ages 13–19; results were largely similar, providing additional support for the use of a composite, cumulative measure (see Supplementary Materials).

As expected, internal consistency was lower for friends’ empathic support provision (Cronbach’s *α* = .44), consistent with the fact that approximately 60% of teens brought a different friend at each wave and thus the friend score reflects empathic behavior sampled across distinct relationships rather than a stable interpersonal trait. Table 1c documents the variability in friendship partners across waves, and descriptive statistics and correlations for all focal variables are presented in Tables 1a and 1b. We further examined gender and family income as predictors of teens’ empathic support provision. Results indicated that both family income (estimate = –0.06, SE = 0.03, *p* = .026) and gender (estimate = 0.21, SE = 0.07, *p* = .006) were associated with teens’ overall empathic support provision toward friends, such that participants with lower baseline family income and female participants demonstrated higher empathic support provision scores across adolescence.

**Table 1a.** Descriptive statistics and correlations among teens’ empathic support provision

Variable	*n*	*M (SD)*	1	2	3	4	5	6	7	8	9
1. Empathic support, age 13	74	2.20 (0.55)	-								
2. Empathic support, age 14	68	2.33 (0.48)	.33**	-							
3. Empathic support, age 15	63	2.33 (0.51)	.42***	.35*	-						
4. Empathic support, age 16	61	2.24 (0.58)	.33*	.16	.54**	-					
5. Empathic support, age 17	70	2.29 (0.50)	.22	.28*	.43**	.55**	-				
6. Empathic support, age 18	62	2.28 (0.59)	.16	.14	.42**	.18	.30*	-			
7. Empathic support, age 19	47	2.46 (0.55)	.26	.23	.37*	.33*	.18	.31*	-		
8. Empathic support, age 21	28	2.27 (0.65)	-.20	.31	.18	.39	.45*	-.26	.14	-	
9. Gender	77	-	.15	.32**	.29*	.16	.18	.06	.06	-.10	-
10. Family income	77	97.43 (1.36)	-.01	-.15	-.27*	-.35**	-.05	-.07	-.16	-.37	.02

*Note:* Gender is coded such that 1 = male, 2 = female. *p < .05, **p < .01.

**Table 1b.** Descriptive statistics and correlations among friends’ empathic support provision

Variable	*n*	*M (SD)*	1	2	3	4	5	6	7	8	9
1. Friend’s support, age 13	75	2.46 (0.72)	-								
2. Friend’s support, age 14	71	2.19 (0.59)	.28*	-							
3. Friend’s support, age 15	67	2.34 (0.50)	.26*	.28*	-						
4. Friend’s support, age 16	65	2.39 (0.61)	-.03	.17	-.19	-					
5. Friend’s support, age 17	71	2.60 (0.54)	-.07	.17	.16	.09	-				
6. Friend’s support, age 18	61	2.50 (0.62)	.03	-.06	.20	-.11	.13	-			
7. Friend’s support, age 19	61	2.62 (0.61)	-.00	.04	.17	-.07	.02	.40**	-		
8. Friend’s support, age 21	65	2.81 (0.52)	-.19	.17	.27*	-.00	.35**	.20	.12	-	

*Note:* Gender is coded such that 1 = male, 2 = female. *p < .05, **p < .01.

**Table 1c.** Characteristics of friends participating in the supportive behavior task across adolescence

Age of supportive behavior task	Percentage of same friends from previous wave	Length of friendship in years *M (SD)*	Closeness of friendship *M* (*SD*)
Age 13	n/a	3.93 (2.88)	4.63 (0.62)
Age 14	34.3%	3.81 (2.83)	4.49 (0.81)
Age 15	48.5%	4.52 (3.09)	4.53 (0.63)
Age 16	43.9%	5.54 (4.14)	4.52 (0.71)
Age 17	43.9%	6.01 (4.21)	4.46 (0.73)
Age 18	45.3%	6.64 (4.08)	4.55 (0.81)
Age 19	28.6%	6.94 (4.65)	4.50 (0.76)
Age 21	28.6%	7.67 (5.25)	4.42 (0.91)

### Main effects of exclusion > inclusion

Main effects of the exclusion > inclusion contrast have been reported elsewhere (Gonzalez et al., 2021). Briefly, we observed increased BOLD signal in dACC, vACC, and right insula for the exclusion > inclusion contrast.

### Does adolescent empathic support provision associate with neural activation of social rejection in early adulthood?

**Figure 1.**
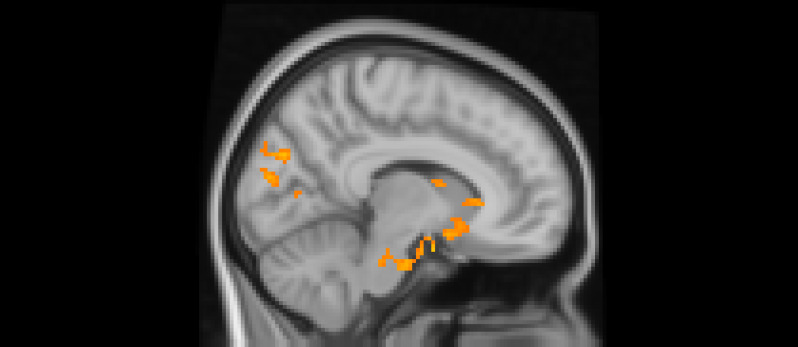
The subgenual cingulate area (*x* = -2, *y* = 10, *z* = 4) and occipital pole (*x* = −4, *y* = −100, *z* = 16). Participants who provided greater empathic support to friends in adolescence showed reduced activity in these clusters when being excluded, compared to when they are being included in ball-tossing game, before adjusting for gender and baseline family income.

Using whole-brain corrected (*z* = 2.3, *p* = .05) covariate analysis, we examined the association between teens’ empathic support *provision* to close peers throughout adolescence and main effects of experiencing exclusion > inclusion in Cyberball task. Clusters were defined by structural probability maps in FSL and EBRAINS and by the continuity of functional activation. Results revealed that higher empathic support provision across adolescence was associated with reduced activation in a cluster centered on the subgenual cingulate area (*x* = −2, *y* = 10, *z* = 4; *p* < .001; Figure 1), extending into adjacent subcortical and paralimbic regions, including subcortical gray matter and the anterior parahippocampal gyrus (Table 2). In addition, a separate posterior cluster showed reduced activation associated with higher empathic support provision, encompassing the occipital pole (*x* = −4, *y* = −100, *z* = 16; *p* = .002) and extending into the cuneal cortex, lingual gyrus, and intracalcarine cortex. Multiple nearby peaks within each cluster were observed, indicating spatially consistent effects across ventral affective and posterior visual processing regions during exclusion relative to inclusion trials.

**Table 2.** Significant clusters of activity for the exclusion > inclusion contrast with teens’ empathic support provision index score before adjusting for gender and baseline family income

MNI coordinates					
Structural location	Cluster size in voxels	*Z*-max	*X*	*Y*	*Z*
Exclusion > inclusion cluster	1598				
Subgenual cingulate area		4.69	–2	10	4
		4.57	–4	10	–16
		4.31	–8	8	–16
		4.21	4	10	4
Subcortical gray matter		4.18	0	–4	–10
Parahippocampal gyrus, anterior division		3.85	12	–4	–24
	673				
Occipital pole		3.92	–4	–100	16
Cuneal cortex		3.51	10	–78	28
Lingual gyrus		3.5	6	–68	4
Intracalcarine cortex		3.5	16	–82	10
Occipital pole		3.43	–6	–104	10
Occipital pole		3.34	–12	–100	10

*Note: X*, *Y*, and *Z* represent coordinates in relation to an origin, usually the anterior commissure, which specifies the coordinates of *X* = 0, *Y* = 0, and *Z* = 0. The *x*-axis goes towards the right side of the brain, the *y*-axis goes towards the front of the brain, and the *z*-axis goes towards the top of the brain. The orientation indicates which direction relative to the origin is positive or negative.

**Figure 2.**
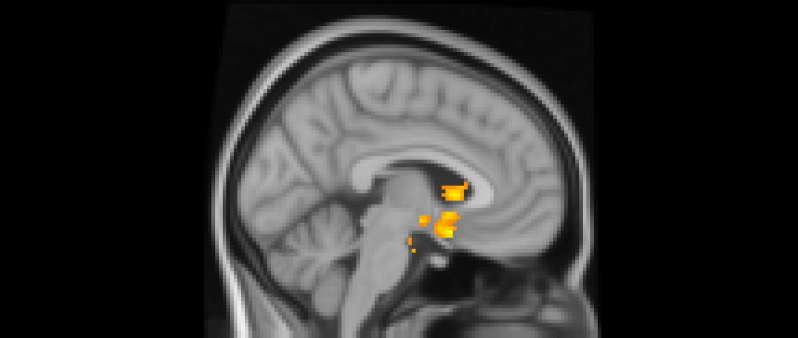
The subgenual cingulate area (*x* = −4, *y* = 10, *z* = −16). Participants who provided greater empathic support to friends in adolescence showed reduced activity in this cluster when being excluded, compared to when they are being included in ball-tossing game, after adjusting for gender and baseline family income.

After adjusting for gender and baseline family income, activation in the subgenual area remained significant (*x* = −4, *y* = 10, *z* = −16; *p* < .001; Figure 2). A nearby peak was also observed within the same cluster at (*x* = –8, *y* = 8, *z* = –16), confirming the robustness of this finding within the subgenual region. Additional peaks of reduced activation associated with higher empathic support scores were identified within striatal and subcortical regions, including the right pallidum/putamen, left nucleus accumbens(NAc), right NAc, and left lateral ventricle, contiguous with subgenual and ventral striatal regions (Table 3).

**Table 3.** Significant clusters of activity for the exclusion > inclusion contrast with teens’ empathic support provision index score after adjusting for gender and baseline family income

MNI coordinates					
Structural location	Cluster size in voxels	*Z*-max	*X*	*Y*	*Z*
Exclusion > Inclusion cluster	1206				
Subgenual cingulate area		4.4	–4	10	–16
Subgenual cingulate area		4.38	–8	8	–16
Left lateral ventricle		4.26	–4	12	4
Right pallidum/putamen		4.22	0	–4	–10
Left accumbens		3.98	–6	8	–8
Right accumbens		3.81	8	10	–6

*Note: X*, *Y*, and *Z* represent coordinates in relation to an origin, usually the anterior commissure, which specifies the coordinates of *X* = 0, *Y* = 0, and *Z* = 0. The *x*-axis goes towards the right side of the brain, the *y*-axis goes towards the front of the brain, and the *z*-axis goes towards the top of the brain. The orientation indicates which direction relative to the origin is positive or negative.

**Figure 3.**
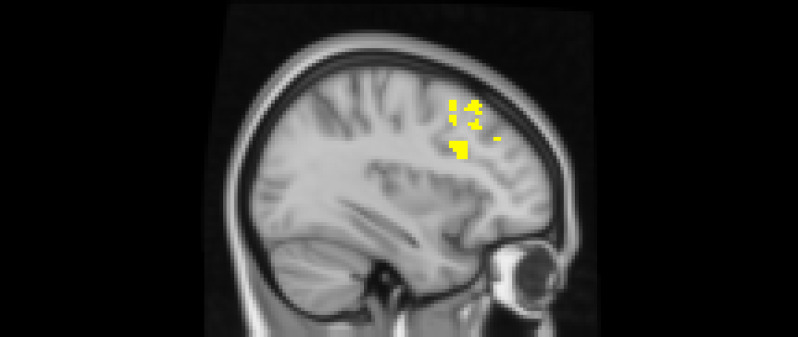
The middle frontal gyrus (*x* = –34, *y* = 24, *z* = 52). Participants who received greater empathic support from friends during adolescence showed reduced activity in the left middle frontal gyrus when being excluded, compared to when they are being included in ball-tossing game. This cluster remained after adjusting for gender and baseline family income.

Using whole-brain corrected (*z* = 2.3, *p* = .05) covariate analysis, we also examined the association between friends’ empathic support provision throughout adolescence and main effects of exclusion in Cyberball task. Results revealed that greater empathic support received from friends across adolescence was associated with reduced activation in the left MFG (*x* = −34, *y* = 24, *z* = 52; *p* = .024; Figure 3, Table 4). After adjusting for gender and baseline family income, pattern of findings remained (left MFG: *x* = –34, *y* = 24, *z* = 52; *p* = .038; Table 5). Several nearby peaks were observed within the same cluster, confirming consistent effects throughout the MFG region. Although the overall pattern of findings was largely consistent across unadjusted and adjusted models, some regional differences emerged; for interpretive clarity and to emphasize effects that account for theoretically relevant covariates, the Discussion focuses on results from the adjusted analyses.

**Table 4.** Significant clusters of activity for the exclusion > inclusion contrast with friends’ empathic support provision index scores before adjusting for gender and baseline family income

MNI coordinates					
Structural location	Cluster size in voxels	*Z*-max	*X*	Y	Z
Exclusion > Inclusion cluster	434				
Middle frontal gyrus		3.95	–34	24	52
		3.66	–42	4	54
		3.56	–38	26	48
		3.52	–42	28	42
		3.34	–34	16	28
		3.26	–32	12	50

*Note: X*, *Y*, and *Z* represent coordinates in relation to an origin, usually the anterior commissure, which specifies the coordinates of *X* = 0, *Y* = 0, and *Z* = 0. The *x*-axis goes towards the right side of the brain, the *y*-axis goes towards the front of the brain, and the *z*-axis goes towards the top of the brain. The orientation indicates which direction relative to the origin is positive or negative.

**Table 5.** Significant clusters of activity for the exclusion > inclusion contrast with friends’ empathic support provision index scores after adjusting for gender and baseline family income

MNI coordinates					
Structural location	Cluster size in voxels	*Z*-max	*X*	*Y*	*Z*
Exclusion > Inclusion cluster	395				
Middle frontal gyrus		3.9	–34	24	52
		3.58	–38	26	48
		3.47	–42	4	54
		3.43	–42	28	42
		3.32	–34	16	28
		3.16	–32	12	50

*Note: X*, *Y*, and *Z* represent coordinates in relation to an origin, usually the anterior commissure, which specifies the coordinates of *X* = 0, *Y* = 0, and *Z* = 0. The *x*-axis goes towards the right side of the brain, the *y*-axis goes towards the front of the brain, and the *z*-axis goes towards the top of the brain. The orientation indicates which direction relative to the origin is positive or negative.

### Does adolescent empathic support provision uniquely associate with adult neural activity to social rejection?

We next focused on two ROIs – subgenual anterior cingulate cortex (sACC) and MFG. These two regions are respectively associated with significant clusters tied to teens’ empathy and friends’ support provision in previous covariate analyses. The subgenual cingulate area was functionally defined using metanalytic capabilities from neurosynth.org by searching the term “subgenual.” Reverse inference statistical map was extracted at the FDR corrected *p* < .01 level. As neurosynth.org does not currently contain MFG map, we used the anatomical Harvard Oxford Cortical and Subcortical atlases to generate MFG mask. Then, both masks were binarized using FSL’s cluster command and the value of neural activity was extracted by applying the sACC and MFG masks to the *Z* scores of the first-level exclusion > inclusion contrast.

To explore the dyadic process of teens’ and friends’ empathic support provision in adolescence and neural response to rejection, we entered the average of teens’ empathic support and friends’ empathic support provision scores across adolescence as separate predictors, and neural activity in response to social rejection at MFG as outcome into a regression model using the *lavaan* package in R (Rosseel, 2012). Neither teens’ empathic support provision (estimate = −0.24, SE = 0.23, *p* = .289) nor friends’ empathic support provision (estimate = −0.16, SE = 0.24, *p* = .510) was associated with neural response to social rejection in the MFG, accounting for their covariance (estimate = 0.04, SE = 0.01, *p* < .001). After adjusting for gender and baseline income, results remained (teens’ empathic support provision: estimate = -0.21, SE = 0.23, *p* = .372; friends’ empathic support provision: estimate = -0.17, SE = 0.24, *p* = .479).

Next, we entered teens’ and friends’ empathic support provision across adolescence and neural response to social rejection at the sACC into a regression model. Results showed that teens’ higher empathic support toward peers during adolescence was linked with reduced neural response to social rejection in the sACC in early adulthood (estimate = −0.54, SE = 0.20, *p* = .006), accounting for the covariance between teens’ and friends’ empathic support provision (estimate = 0.04, SE = 0.01, *p* < .001). On the other hand, friends’ empathic support provision did not significantly associate with neural response to rejection at the sACC (estimate = −0.06, SE = 0.21, *p* = .789). After adjusting for gender and baseline income, results remained (teen’s empathic support provision: estimate = -0.55, SE = 0.20, *p* = .007; friends’ empathic support provision: estimate = -.05, SE = .21, *p* = .799).

### Follow-up analysis: does felt distress mediate the link between adolescent empathy and sACC activation?

Lastly, to examine whether subjective social distress during exclusion mediated the association between adolescent empathic support provision and sACC reactivity to social rejection, we conducted a follow-up mediation analysis using maximum likelihood estimation with 5,000 bootstrap draws, using the *lavaan* package in *R* (Rosseel, 2012). Specifically, we tested whether Need Threat Scale (NTS) scores during Cyberball exclusion > inclusion trials mediated the link between adolescents’ empathic support provision (ages 13 to 21) and sACC activation in early adulthood.

Prior to analysis, NTS scores were mean-centered to facilitate model interpretation. Results showed that greater adolescent empathic support provision was significantly associated with reduced sACC activation during social rejection in early adulthood (*β* = −0.55, SE = 0.18, z = −3.08, *p* = .002), indicating a direct association between adolescents’ empathic support toward peers and attenuated neural sensitivity to social threat in adulthood. The path from teens’ empathic support provision to self-reported social distress during exclusion (NTS) was positive but not statistically significant (*β* = 3.71, SE = 2.76, *z* = 1.35, *p* = .179). In turn, NTS was not significantly associated with sACC activation (*β* = −0.002, SE = 0.007, *z* = −0.34, *p* = .731).

Consistent with these findings, the indirect effect of adolescent empathic support provision on sACC activation via NTS was not significant (*β* = −0.01, SE = 0.03, *z* = −0.27, *p* = .785), indicating that adolescents’ subjective experience of social distress during exclusion did not mediate the association between empathic support provision and adult neural responses to rejection although the total effect of adolescent empathic support provision on sACC activation remained significant (*β* = −0.56, SE = 0.18, *z* = −3.21, *p* = .001). Results remained after adjusting for gender and baseline family income (indirect: *β* = −0.01, SE = 0.03, *z* = −0.24, *p* = .812; direct: *β* = −0.58, SE = 0.19, *z* = −3.02, *p* = .003). Taken together, these results suggest that the association between adolescent empathic support provision and reduced sACC reactivity to social rejection in early adulthood is largely independent of participants’ consciously reported social distress during the exclusion task.

## Discussion

The present study examined whether teens’ empathic support provision was linked to adult relationship functioning in terms of young adults’ neural responses to social rejection. Employing longitudinal observational data collected from ages 13 to 21 and fMRI data from the Cyberball paradigm in early adulthood (age 24), we found that greater empathic support provision toward close friends during adolescence was associated with reduced neural activation in the sACC during social exclusion in early adulthood. This pattern remained even after accounting for empathic support received from close friends. These findings contribute to an increasing body of research examining developmental roots of the adult social brain. Our results built on prior work by demonstrating that empathy – a key social skill emerging across adolescence – has long-term neural implications for how individuals respond to rejection-related social events in early adulthood.

It is important to note that although our measure was designed to capture empathic support provision, our coding system primarily reflects cognitive components of empathy. Specifically, dimensions such as interpretation of the friend’s problem, instrumental support provision, and engagement emphasize the adolescent’s capacity to understand, reason about, and respond appropriately to another’s perspective or needs – hallmarks of cognitive empathy. While some elements, such as emotional support provision incorporate affective attunement, these behaviors are still coded in terms of the participant’s observable responsiveness and understanding rather than their internal emotional resonance with the friend’s distress. Thus, our construct of empathic support reflects a form of empathy oriented toward perspective taking and problem understanding rather than shared affect. This may explain why our findings suggest that empathic support functioned as a protective factor against rejection sensitivity as cognitive empathy may enable adolescents to interpret social cues more accurately and regulate responses to interpersonal stress, consistent with prior findings (Tan et al., 2023). Future research should employ measures that capture affective empathy and test how distinct components of empathy heighten vulnerability or protect teens against social rejection.

The sACC, the most ventral portion of the ACC, is increasingly recognized as a key neural hub in processing social rejection. Evidence from fMRI studies has shown that the sACC is consistently activated in response to social exclusion across various social rejection tasks (e.g., Masten et al., 2009; Sebastian et al., 2011), even when other aspects of the task are controlled for (i.e., expectancy violation; Bolling et al., 2011). The present findings extend this literature by demonstrating that greater empathic support provision during adolescence prospectively predicts reduced sACC activation during social exclusion in early adulthood, underscoring the relevance of this region for understanding long-term sensitivity to social threat. Notably, self-reported social distress, as indexed by the Need Threat Scale (NTS), did not mediate the association between adolescent empathic support provision and sACC activation during social exclusion. This absence of mediation suggests that the observed neural effects are not solely attributable to momentary, consciously reported distress during exclusion. Rather, the findings point to broader neural processes through which adolescent empathic experiences may shape adult responses to social threat.

Accumulating evidence indicates that the sACC indexes not only subjective affective distress but also regulatory, monitoring, and integrative functions during socially salient experiences. The sACC has been implicated in modulating negative affect, coordinating autonomic responses, and integrating cognitive–affective signals in the context of social threat (Alexander et al., 2020; Dixon et al., 2017; Mayberg, 2003). Thus, although the present study does not include a direct measure of self-regulation, one plausible interpretation is that adolescents who consistently provide empathic support develop neural profiles characterized by altered engagement of ventromedial regulatory systems when confronted with social rejection. Importantly, this regulatory interpretation may operate in at least two distinct directions.

On the one hand, sACC activation may reflect a reduced need to recruit regulatory resources during social exclusion. From this perspective, adolescents who show higher empathic support provision may be more resilient to social threat, through reduced need for self-regulation in response to rejection. This interpretation is consistent with prior work showing that lower neighborhood quality in adolescence predicted heightened dorsal ACC activation during social exclusion in adulthood, without a corresponding increase in self-reported distress, a pattern interpreted as reflecting heightened social vigilance or monitoring rather than subjective distress per se (Gonzalez et al., 2015). Similarly, experimental work demonstrating that supportive physical contact attenuates threat-related neural responses suggests that social support can reduce the need to mobilize regulatory resources in the face of perceived threat in adulthood (Coan et al., 2017).

On the other hand, reduced sACC activation may simply reflect diminished engagement of regulatory processes during exclusion rather than reduced regulatory demand. Prior work has emphasized the role of the sACC and related social pain circuitry as markers of affective vulnerability. For example, Masten et al. (2009) found that greater sACC activation during social exclusion was associated with higher subjective distress, whereas greater engagement of lateral prefrontal regions implicated in regulatory control was associated with lower distress and reduced sACC activity. Within this framework, reduced sACC activation may reflect less recruitment of regulatory resources, rather than more efficient regulation.

A third possibility is methodological: The present analyses tested mediation effects, which impose additional statistical constraints, and the observed neural effects may be relatively small in magnitude. As such, limited power to detect indirect effects, coupled with restricted variability in self-reported distress, may have reduced sensitivity to mediation via the NTS. Future studies that directly assess regulatory or other underlying processes using concurrent physiological indices (e.g., heart rate variability, skin conductance), behavioral markers, or task-based measures will help clarify whether adolescent empathic support provision confers resilience by reducing the need for regulation, enhances regulatory capacity, or alters sensitivity to social threat in ways not captured by self-report.

Developmental neuroimaging research further underscores that the sACC exhibits heightened sensitivity to social exclusion during adolescence relative to adulthood (Masten et al., 2009). Past research showed that children (Rotge et al., 2015) and adolescents (Bolling et al., 2011) displayed unique ventral ACC activity during exclusion than adult participants. Moreover, this sensitivity appears modulated by pubertal development, with adolescents in more advanced stages of puberty exhibiting greater neural responses to peer rejection (Silk et al., 2014). These developmental shifts in sACC responsivity are thought to be linked with increased susceptibility to affective dysregulation, as they coincide with heightened importance of peer relationships and socio-emotional self-evaluation. Our study extends this developmental framework by showing that early empathic abilities may be linked to the functional maturation of the sACC, contributing to reduced reactivity to social threat in early adulthood. Interventions promoting prosocial engagement during adolescence could contribute to long-term regulatory recalibration of sACC responsivity and support other mental functions across the transition from adolescence into adulthood.

Beyond the sACC, the current study also identified reduced activation in striatal regions, including the right putamen and bilateral NAc, among young adults who displayed higher empathic support provision during adolescence. This attenuation may reflect more adaptive affective calibration, in which rejection cues are integrated with less disruption to reward-related processes. Supporting this view, Smith and colleagues (2021) demonstrated that the ACC and its projections to the NAc are causally involved in the social transfer of both pain and analgesia in rodents, indicating that ACC–NAc circuits may serve as a key pathway through which empathy-related processes influence motivational and affective states. Similarly, smaller putamen volume and right cortical infarction were associated with impaired empathy following subacute ischemic stroke, underscoring the putamen’s contribution to the neural underpinnings of empathic and social functioning (Qu et al., 2022). Reduced activation in these regions during social exclusion may indicate a diminished affective salience of social rejection among individuals with greater empathic engagement, possibly reflecting decreased motivational relevance associated with social loss.

Our results also suggest that empathic support received from close friends during adolescence is associated with reduced activation in the middle frontal gyrus (MFG) during social rejection in early adulthood. The MFG, situated between the superior and inferior frontal gyri, is a core component of the lateral prefrontal cortex and a key node of the cognitive control network involved in higher-order executive functions (Li et al., 2013), including socioemotional understanding and self-regulation (Schmidt et al., 2017). Our findings align with prior research showing that individuals who receive support from close others during threat anticipation show reduced activation in prefrontal regions, suggesting that supportive social presence dampens the need for effortful top-down regulation (Coan et al., 2017). Importantly, conflicting interpretations should also be considered. Prior work has linked higher prefrontal activation with greater regulatory engagement (Eisenberger et al., 2003; Masten et al., 2009). In contrast, in the present study, higher friend-provided empathic support provision during adolescence was associated with lower MFG activation, which could reflect lower engagement in cognitive control resources, rather than more efficient regulation. Notably, the low internal consistency of the friend-support composite, likely due to variation in friends across waves, warrants cautious interpretation.

Importantly, when both adolescent and friend’s empathic support were entered into the same model, only teens’ own empathic support provision was significantly associated with sACC reactivity to social rejection while the effect of friends’ empathic support on MFG activation was no longer significant. This finding suggests that the long-term neural association of *giving* empathic support to close others may be more robust than the impact of receiving empathic support. This pattern indicates that the shared variance between empathic support provision and receipt may reflect broader traits of social competence. Conceptually, this finding aligns with emerging research suggesting that the act of empathic support provision is more strongly tied to affective resilience (Xing et al., 2023), as it engages systems involved in caregiving and emotion regulation (Stern et al., 2021). In contrast, the benefits of receiving support – though important – may be more context-dependent and externally scaffolded, and less associative of stable changes in intrinsic neural functioning over time.

The relevance of our findings extends beyond social neuroscience and into the clinical domain, as they identify a potential protective mechanism linking adolescent empathy to neural systems implicated in depression. The sACC has been implicated as a central neural marker in the development and maintenance of major depressive disorder (Silk et al., 2023). The sACC is centrally implicated in the neurobiology of major depressive disorder, with abnormalities in its structure, function, and connectivity consistently observed in both clinical and subclinical populations (Drevets et al., 1997; Hamani et al., 2011). Social rejection, particularly during formative years, is a potent precipitant of depressive symptoms (Kendler et al., 2003). Several studies have shown that heightened sACC response to exclusion, particularly in paradigms such as Cyberball, predicts future increases in depressive symptoms among adolescents (Jankowski et al., 2018; Silk et al., 2023). Notably, the sACC is thought to mediate physiological arousal (e.g., vagal tone, heart rate variability, cortisol response; Dixon et al., 2017; Mayberg, 2003), potentially explaining how heightened sensitivity to interpersonal stressors may lead to affective and somatic symptoms tied to psychopathology. Our findings enrich this framework, suggesting that adolescents with greater empathy may be protected neurally from rejection-induced emotional dysregulation in early adulthood. Reduced sACC reactivity may indicate a lower allostatic load and greater social-emotional resilience, providing a promising neurobiological mechanism through which adolescent social skills may buffer against later psychopathology.

### Limitations and future directions

We acknowledge several study limitations. First, although the use of a longitudinal, observational design strengthens ecological validity, the final fMRI sample size was modest due to attrition and imaging constraints with effect sizes ranging from very small to small, which limits the generalizability of our findings. Additionally, our sample size did not provide sufficient power to examine potential gender differences in empathic support provision or its longitudinal associations with rejection sensitivity. Future research with larger samples will be critical for replicating these findings and testing whether the protective effects of empathic support on rejection sensitivity operate similarly across genders or reflect gender-specific socialization processes.

Although our findings demonstrate that empathic support provision during adolescence may buffer against neural sensitivity to social rejection in early adulthood, we cannot conclusively demonstrate that empathy is shaping neural responses without repeated measures of brain activity or experimental manipulations of empathy. Neither can we rule out the reverse causal pathway whereby unmeasured adolescent neural processes influence empathy, producing the observed association. Longitudinal designs incorporating repeated neuroimaging and multi-method assessments of empathy could help disentangle these temporal dynamics.

Third, while we assessed empathic support provision from both teens and their friends annually from ages 13 to 21, these observational measures cannot fully capture the nuances of adolescents’ broader social experiences or relational dynamics outside of the laboratory. Incorporating ecological momentary assessment or digital tracking devices may offer complementary insights into how support behaviors function across contexts. Likewise, while the Cyberball paradigm is a widely used method for eliciting neural responses to social rejection, it cannot fully capture the complexity of real-world exclusion experiences. Future work should consider multimodal paradigms that simulate more personalized rejection scenarios to better link neural activity with psychological outcomes.

Finally, although our findings suggest that early empathic support provision may buffer against heightened neural sensitivity to social rejection, we did not directly examine the mechanisms through which this effect may occur. Future research should explore other potential developmental mediators (i.e., emotion regulation) that may help explain the pathway from adolescent empathy to adult neural responses to rejection. Moreover, current evidence from attachment theory (Bowlby, 1969; Stern et al., 2021) suggests that attachment security may play a critical role in shaping both empathic caregiving and sensitivity to social threat. Although our study did not assess attachment, future work should examine whether early attachment orientations influence the development of empathic support provision and its neural correlates with rejection sensitivity, offering a broader framework for understanding individual differences in social-emotional resilience. Additionally, we did not analyze adult mental health outcomes such as depression, anxiety, or social functioning. Future work should investigate whether reduced sACC reactivity serves as a neurobiological risk factor linking adolescent empathy to long-term clinical outcomes, particularly in individuals at elevated risk for affective disorders.

### Conclusion

The present study provides novel longitudinal evidence linking adolescent empathic support provision to neural responses to social rejection in early adulthood. Our findings suggest that early-developed empathy skills within close friendships may forecast the reactivity of the sACC, a key neural hub implicated in affective regulation and modulation. Empathic support received from peers appears to be linked to the MFG, although this pattern was not significant after accounting for the covariance with teens’ empathic support provision. Additional analyses further revealed that this pattern was not mediated by participants’ self-reported felt distress during the exclusion task, as measured by the Need Threat Scale (NTS), suggesting that the long-term effects of adolescent empathy on the sACC response to social rejection may operate independently of conscious emotional experience in the moment. Together, these findings highlight the potential developmental pathway from adolescent peer experiences to neural correlates and the potential developmental pathway from empathic support provision to resilience. By linking normative variations in adolescent empathy to the sACC function, an area centrally implicated in mood disorders and social rejection, this work bridges social developmental neuroscience with affective vulnerability. Reduced sACC reactivity among more empathic adolescents may reflect neural tuning that buffers against future emotional dysregulation. Ultimately, this work offers a framework for understanding how early interpersonal behaviors confer lasting neural adaptations that may protect against social stress and future psychopathology.

## Supporting information

10.1017/S095457942610131X.sm001 Lin et al. supplementary material Lin et al. supplementary material

## Data Availability

Participants’ data are protected by a Confidentiality Certificate issued by the U.S. Department of Health and Human Services. Method, materials, and analysis code are available on https://osf.io/zx5vg/.

## Supplementary material

The supplementary material for this article can be found at https://doi.org/10.1017/S095457942610131X.

## Preregistration statement

The present study involves secondary analyses of an existing longitudinal dataset. The analysis plan was preregistered prior to conducting the analyses reported in this manuscript and publicly accessible at: https://osf.io/zx5vg/ (March 24^th^, 2025). In response to mixed findings in the prior literature regarding the direction of empathy-related neural effects, the present manuscript treats all neural associations as exploratory and directionally unspecified.

## References

[ref1] Alexander, L. , Wood, C. M. , Gaskin, P. L. R. , Sawiak, S. J. , Fryer, T. D. , Hong, Y. T. , McIver, L. , Clarke, H. F. , & Roberts, A. C. (2020). Over-activation of primate subgenual cingulate cortex enhances the cardiovascular, behavioral and neural responses to threat. Nature Communications, 11(1), 5386. 10.1038/s41467-020-19167-0 PMC7588412 33106488

[ref2] Allemand, M. , Steiger, A. E. , & Fend, H. A. (2015). Empathy development in adolescence predicts social competencies in adulthood. Journal of Personality, 83(2), 229–241. 10.1111/jopy.12098 24684661

[ref3] Allen, J. P. , Hall, F. D. , Insabella, G. M. , Land, D. J. , Marsh, P. A. , & Porter, M. R. (2001). *Supportive behavior coding system.* Supportive behavior coding system. Unpublished manuscript. University of Virginia.

[ref4] Allen, J. P. , Narr, R. K. , Kansky, J. , & Szwedo, D. E. (2020). Adolescent peer relationship qualities as predictors of long-term romantic life satisfaction. Child Development, 91(1), 327–340. 10.1111/cdev.13193 30675714 PMC6656620

[ref5] Araiza, A. M. , Freitas, A. L. , & Klein, D. N. (2020). Social-experience and temperamental predictors of rejection sensitivity: A prospective study. Social Psychological and Personality Science, 11(6), 733–742. 10.1177/1948550619878422

[ref6] Asscheman, J. S. , Koot, S. , Ma, I. , Marieke Buil, J. , Krabbendam, L. , Cillessen, A. H. N. , & van Lier, P. A. C. (2020). Heightened neural sensitivity to social exclusion in boys with a history of low peer preference during primary school. Developmental Cognitive Neuroscience, 44, 100792. 10.1016/j.dcn.2020.100792 32716848 PMC7374540

[ref7] Beyer, F. , Münte, T. F. , & Krämer, U. M. (2014). Increased neural reactivity to socio-emotional stimuli links social exclusion and aggression. Biological Psychology, 96, 102–110. 10.1016/j.biopsycho.2013.12.008 24368143

[ref8] Bolling, D. Z. , Pitskel, N. B. , Deen, B. , Crowley, M. J. , Mayes, L. C. , & Pelphrey, K. A. (2011). Development of neural systems for processing social exclusion from childhood to adolescence. Developmental Science, 14(6), 1431–1444. 10.1111/j.1467-7687.2011.01087.x 22010901 PMC4457505

[ref9] Bowlby, J. (1969). Attachment and Loss, Vol. 1: Attachment. Basic Books.

[ref10] Chester, D. S. , & DeWall, C. N. (2014). Prefrontal recruitment during social rejection predicts greater subsequent self-regulatory imbalance and impairment: Neural and longitudinal evidence. NeuroImage, 101, 485–493. 10.1016/j.neuroimage.2014.07.054 25094019 PMC4170689

[ref11] Cicchetti, D. V. , & Sparrow, S. A. (1981). Developing criteria for establishing interrater reliability of specific items: Applications to assessment of adaptive behavior. American Journal of Mental Deficiency, 86(2), 127–137. 7315877

[ref12] Coan, J. A. , Beckes, L. , Gonzalez, M. Z. , Maresh, E. L. , Brown, C. L. , & Hasselmo, K. (2017). Relationship status and perceived support in the social regulation of neural responses to threat. Social Cognitive and Affective Neuroscience, 12(10), 1574–1583. 10.1093/scan/nsx091 28985422 PMC5647795

[ref13] Costello, M. A. , Allen, J. P. , Womack, S. R. , Loeb, E. L. , Stern, J. A. , & Pettit, C. (2023). Characterizing emotional support development: From adolescent best friendships to young adult romantic relationships. Journal of Research on Adolescence, 33(2), 389–403. 36305166 10.1111/jora.12809 PMC10140188

[ref14] Dixon, M. L. , Thiruchselvam, R. , Todd, R. , & Christoff, K. (2017). Emotion and the prefrontal cortex: An integrative review. Psychological Bulletin, 143(10), 1033–1081. 10.1037/bul0000096 28616997

[ref15] Downey, G. , & Feldman, S. I. (1996). Implications of rejection sensitivity for intimate relationships. Journal of Personality & Social Psychology, 70(6), 1327–1343. 10.1037/0022-3514.70.6.1327 8667172

[ref16] Downey, G. , Lebolt, A. , Rincón, C. , & Freitas, A. L. (1998). Rejection sensitivity and children’s interpersonal difficulties. Child Development, 69(4), 1074–1091. 9768487

[ref17] Drevets, W. C. , Price, J. L. , Simpson, J. R., Jr. , Todd, R. D. , Reich, T. , Vannier, M. , & Raichle, M. E. (1997). Subgenual prefrontal cortex abnormalities in mood disorders. Nature, 386(6627), 824–827. 10.1038/386824a0 9126739

[ref18] Eisenberger, N. I. , Lieberman, M. D. , & Williams, K. D. (2003). Does rejection hurt? An FMRI study of social exclusion. Science, 302(5643), 290–292. 10.1126/science.1089134 14551436

[ref19] Fan, M. , Jie, J. , Luo, P. , Pang, Y. , Xu, D. , Yu, G. , Zhao, S. , Chen, W. , & Zheng, X. (2021). Social exclusion down-regulates pain empathy at the late stage of empathic responses: Electrophysiological evidence. Frontiers in Human Neuroscience, 15, 634714. 10.3389/fnhum.2021.634714 33732123 PMC7956954

[ref20] Feldman, S. , & Downey, G. (1994). Rejection sensitivity as a mediator of the impact of childhood exposure to family violence on adult attachment behavior. Development and Psychopathology, 6(1), 231–247. 10.1017/S0954579400005976 23438329

[ref21] Field, N. H. , Balkind, E. , Burnell, K. , Fox, K. A. , Feldman, M. J. , Nick, E. A. , Telzer, E. H. , Lindquist, K. A. , & Prinstein, M. J. (2025). Popularity, but not likability, as a risk factor for low empathy: A longitudinal examination of within- and between-person effects of peer status and empathy in adolescence. Developmental Psychology, 61(9), 1684–1697. 10.1037/dev0001914 39818918 PMC13005297

[ref22] Gao, S. , Assink, M. , Bi, C. , & Chan, K. L. (2024). Child maltreatment as a risk factor for rejection sensitivity: A three-level meta-analytic review. Trauma, Violence & Abuse, 25(1), 680–690. 10.1177/15248380231162979 37036152

[ref23] Gao, S. , Assink, M. , Cipriani, A. , & Lin, K. (2017). Associations between rejection sensitivity and mental health outcomes: A meta-analytic review. Clinical Psychology Review, 57, 59–74. 10.1016/j.cpr.2017.08.007 28841457

[ref24] Gonzalez, M. Z. , Beckes, L. , Chango, J. , Allen, J. P. , & Coan, J. A. (2015). Adolescent neighborhood quality predicts adult dACC response to social exclusion. Social Cognitive and Affective Neuroscience, 10(7), 921–928. 10.1093/scan/nsu137 25349459 PMC4483560

[ref25] Gonzalez, M. Z. , Coppola, A. M. , Allen, J. P. , & Coan, J. A. (2021). Yielding to social presence as a bioenergetic strategy. Current Research in Ecological and Social Psychology, 2, 100010. 10.1016/j.cresp.2021.100010 35187511 PMC8851505

[ref26] Hamani, C. , Mayberg, H. , Stone, S. , Laxton, A. , Haber, S. , & Lozano, A. M. (2011). The subcallosal cingulate gyrus in the context of major depression. Biological Psychiatry, 69(4), 301–308. 10.1016/j.biopsych.2010.09.034 21145043

[ref27] Jamieson, J. P. , Harkins, S. G. , & Williams, K. D. (2010). Need threat can motivate performance after ostracism. Personality & Social Psychology Bulletin, 36(5), 690–702. 10.1177/0146167209358882 20388870

[ref28] Jankowski, K. F. , Batres, J. , Scott, H. , Smyda, G. , Pfeifer, J. H. , & Quevedo, K. (2018). Feeling left out: Depressed adolescents may atypically recruit emotional salience and regulation networks during social exclusion. Social Cognitive and Affective Neuroscience, 13(8), 863–876. 10.1093/scan/nsy055 30059994 PMC6123522

[ref29] Jaworska, N. , & MacQueen, G. (2015). Adolescence as a unique developmental period. Journal of Psychiatry & Neuroscience, 40(5), 291–293. 10.1503/jpn.150268 26290063 PMC4543091

[ref30] Jenkinson, M. , Bannister, P. , Brady, M. , & Smith, S. (2002). Improved optimization for the robust and accurate linear registration and motion correction of brain images. NeuroImage, 17, 825–841. 10.1016/s1053-8119(02)91132-8 12377157

[ref31] Kellij, S. , Dobbelaar, S. , Lodder, G. M. A. , Veenstra, R. , & Güroğlu, B. (2024). Here comes revenge: Peer victimization relates to neural and behavioral responses to social exclusion. Research on Child and Adolescent Psychopathology, 52(12), 1913–1930. 10.1007/s10802-024-01227-4 39287772 PMC11624251

[ref32] Kendler, K. S. , Hettema, J. M. , Butera, F. , Gardner, C. O. , & Prescott, C. A. (2003). Life event dimensions of loss, humiliation, entrapment, and danger in the prediction of onsets of major depression and generalized anxiety. Archives of General Psychiatry, 60(8), 789–796. 10.1001/archpsyc.60.8.789 12912762

[ref33] Li, W. , Qin, W. , Liu, H. , Fan, L. , Wang, J. , Jiang, T. , & Yu, C. (2013). Subregions of the human superior frontal gyrus and their connections. NeuroImage, 78, 46–58. 10.1016/j.neuroimage.2013.04.011 23587692

[ref34] Loeb, E. L. , Stern, J. A. , Costello, M. A. , & Allen, J. P. (2021). With(out) a little help from my friends: Insecure attachment in adolescence, support-seeking, and adult negativity and hostility. Attachment & Human Development, 23(5), 624–642. 10.1080/14616734.2020.1821722 32990166 PMC8005498

[ref35] Marston, E. G. , Hare, A. , & Allen, J. P. (2010). Rejection sensitivity in late adolescence: Social and emotional sequelae. Journal of Research on Adolescence, 20(4), 959–982. 10.1111/j.1532-7795.2010.00675.x 21113326 PMC2990973

[ref36] Masten, C. L. , Eisenberger, N. I. , Borofsky, L. A. , Pfeifer, J. H. , McNealy, K. , Mazziotta, J. C. , & Dapretto, M. (2009). Neural correlates of social exclusion during adolescence: Understanding the distress of peer rejection. Social Cognitive and Affective Neuroscience, 4(2), 143–157. 10.1093/scan/nsp007 19470528 PMC2686232

[ref37] Masten, C. L. , Morelli, S. A. , & Eisenberger, N. I. (2011). An fMRI investigation of empathy for social pain’ and subsequent prosocial behavior. NeuroImage, 55(1), 381–388. 10.1016/j.neuroimage.2010.11.060 21122817

[ref38] Masten, C. L. , Telzer, E. H. , Fuligni, A. J. , Lieberman, M. D. , & Eisenberger, N. I. (2012). Time spent with friends in adolescence relates to less neural sensitivity to later peer rejection. Social Cognitive and Affective Neuroscience, 7(1), 106–114. 10.1093/scan/nsq098 21183457 PMC3252626

[ref39] Mayberg, H. S. (2003). Modulating dysfunctional limbic-cortical circuits in depression: Towards development of brain-based algorithms for diagnosis and optimised treatment. British Medical Bulletin, 65, 193–207. 10.1093/bmb/65.1.193 12697626

[ref40] Meyer, M. L. , Masten, C. L. , Ma, Y. , Wang, C. , Shi, Z. , Eisenberger, N. I. , Lieberman, M. D. , & Han, S. (2015). Differential neural activation to friends and strangers links interdependence to empathy. Culture and Brain, 3(1), 21–38. 10.1007/s40167-014-0023-7

[ref41] Onoda, K. , Okamoto, Y. , Nakashima, K. , Nittono, H. , Ura, M. , & Yamawaki, S. (2009). Decreased ventral anterior cingulate cortex activity is associated with reduced social pain during emotional support. Social Neuroscience, 4(5), 443–454. 10.1080/17470910902955884 19562631

[ref42] Paus, T. , Keshavan, M. , & Giedd, J. N. (2008). Why do many psychiatric disorders emerge during adolescence? Nature Reviews Neuroscience, 9(12), 947–957. 10.1038/nrn2513 19002191 PMC2762785

[ref43] Pfefferbaum, A. , Mathalon, D. H. , Sullivan, E. V. , Rawles, J. M. , Zipursky, R. B. , & Lim, K. O. (1994). A quantitative magnetic resonance imaging study of changes in brain morphology from infancy to late adulthood. Archives of Neurology, 51(9), 874–887. 10.1001/archneur.1994.00540210046012 8080387

[ref44] Portt, E. , Person, S. , Person, B. , Rawana, E. , & Brownlee, K. (2020). Empathy and positive aspects of adolescent peer relationships: A scoping review. Journal of Child and Family Studies, 29(9), 2416–2433. 10.1007/s10826-020-01753-x

[ref45] Qu, J. F. , Zhou, Y. Q. , Liu, J. F. , Hu, H. H. , Cheng, W. Y. , Lu, Z. H. , Shi, L. , Luo, Y. S. , Zhao, L. , & Chen, Y. K. (2022). Right cortical infarction and a reduction in putamen volume may be correlated with empathy in patients after subacute ischemic stroke-a multimodal magnetic resonance imaging study. Journal of Clinical Medicine, 11(15), 4479. 10.3390/jcm11154479 35956096 PMC9369598

[ref46] Rosseel, Y. (2012). Lavaan: An R package for structural equation modeling. Journal of Statistical Software, 48(2), 1–36. 10.18637/jss.v048.i02

[ref47] Rotge, J. Y. , Lemogne, C. , Hinfray, S. , Huguet, P. , Grynszpan, O. , Tartour, E. , George, N. , & Fossati, P. (2015). A meta-analysis of the anterior cingulate contribution to social pain. Social Cognitive and Affective Neuroscience, 10(1), 19–27. 10.1093/scan/nsu110 25140048 PMC4994851

[ref48] Schmidt, T. , Roser, P. , Ze, O. , Juckel, G. , Suchan, B. , & Thoma, P. (2017). Cortical thickness and trait empathy in patients and people at high risk for alcohol use disorders. Psychopharmacology, 234(23-24), 3521–3533. 10.1007/s00213-017-4741-3 28971228

[ref49] Sebastian, C. L. , Tan, G. C. , Roiser, J. P. , Viding, E. , Dumontheil, I. , & Blakemore, S. J. (2011). Developmental influences on the neural bases of responses to social rejection: Implications of social neuroscience for education. NeuroImage, 57(3), 686–694. 10.1016/j.neuroimage.2010.09.063 20923708

[ref50] Silk, J. S. , Sequeira, S. S. , Jones, N. P. , Lee, K. H. , Dahl, R. E. , Forbes, E. E. , Ryan, N. D. , & Ladouceur, C. D. (2023). Subgenual anterior cingulate cortex reactivity to rejection Vs. Acceptance predicts depressive symptoms among adolescents with an anxiety history. Journal of Clinical Child and Adolescent Psychology, 52(5), 659–674. 10.1080/15374416.2021.2019048 35072560 PMC9308833

[ref51] Silk, J. S. , Siegle, G. J. , Lee, K. H. , Nelson, E. E. , Stroud, L. R. , & Dahl, R. E. (2014). Increased neural response to peer rejection associated with adolescent depression and pubertal development. Social Cognitive and Affective Neuroscience, 9(11), 1798–1807. 10.1093/scan/nst175 24273075 PMC4221220

[ref52] Smith, M. L. , Asada, N. , & Malenka, R. C. (2021). Anterior cingulate inputs to nucleus accumbens control the social transfer of pain and analgesia. Science, 371(6525), 153–159. 10.1126/science.abe3040 33414216 PMC7952019

[ref53] Smith, S. M. (2002). Fast robust automated brain extraction. Human Brain Mapping, 17(3), 143–155. 10.1002/hbm.10062 12391568 PMC6871816

[ref54] Somerville, L. H. , Heatherton, T. F. , & Kelley, W. M. (2006). Anterior cingulate cortex responds differentially to expectancy violation and social rejection. Nature Neuroscience, 9(8), 1007–1008. 10.1038/nn1728 16819523

[ref55] Steinhoff, A. , & Keller, M. (2020). Pathways from childhood sociomoral sensitivity in friendship, insecurity, and peer rejection to adult friendship quality. Child Development, 91(5), e1012–e1029. 10.1111/cdev.13381 32627194

[ref56] Stern, J. A. , Bailey, N. A. , Costello, M. A. , Hellwig, A. F. , Mitchell, J. , & Allen, J. P. (2024). Empathy across three generations: From maternal and peer support in adolescence to adult parenting and child outcomes. Child Development, 95(5), 1628–1640. 10.1111/cdev.14109 38774980 PMC11576248

[ref57] Stern, J. A. , Costello, M. A. , Kansky, J. , Fowler, C. , Loeb, E. L. , & Allen, J. P. (2021). Here for you: Attachment and the growth of empathic support for friends in adolescence. Child Development, 92(6), e1326–e1341. 10.1111/cdev.13630 34263461 PMC8599634

[ref58] Tan, X. , Yang, Y. , & Yu, M. (2023). Longitudinal relationship of empathy and social anxiety among adolescents: The mediation roles of psychological inflexibility and rejection sensitivity. Journal of Affective Disorders, 339, 867–876. 10.1016/j.jad.2023.07.069 37467804

[ref59] Tavakol, M. , & Dennick, R. (2011). Making sense of Cronbach’s alpha. International Journal of Medical Education, 2, 53–55. 10.5116/ijme.4dfb.8dfd 28029643 PMC4205511

[ref60] Van der Graaff, J. , Branje, S. , De Wied, M. , Hawk, S. , Van Lier, P. , & Meeus, W. (2014). Perspective taking and empathic concern in adolescence: Gender differences in developmental changes. Developmental Psychology, 50, 881–888. 10.1037/a0034325 24040846

[ref61] Will, G. J. , van Lier, P. A. , Crone, E. A. , & Güroğlu, B. (2016). Chronic childhood peer rejection is associated with heightened neural responses to social exclusion during adolescence. Journal of Abnormal Child Psychology, 44(1), 43–55. 10.1007/s10802-015-9983-0 25758671 PMC4715124

[ref62] Williams, K. D. , Cheung, C. K. T. , & Choi, W. (2000). Cyberostracism: Effects of being ignored over the internet. Journal of Personality and Social Psychology, 79(5), 748–762. 10.1037/0022-3514.79.5.748 11079239

[ref63] Xing, L. , Deng, S. W. , & Ho, G. W. (2023). From empathy to resilience: The mediating role of emotional intelligence. Psychological Reports, 128, 4533–4548. 10.1177/00332941231220299 38063027

