# Supplementary material for: Adolescent empathy predicts reduced neural responses to social rejection in adulthood
Source: Dev Psychopathol. 2026 Mar 4:1–13. Online ahead of print. doi: 10.1017/S095457942610131X (PMC12972612; doi:10.1017/S095457942610131X)
Supplement: Lin et al. supplementary material [file S095457942610131Xsup001.docx]

**Supplementary Materials**

**Is Adolescent Empathic Support Provision (Ages 13-19) Associated with Neural Activation of Social Rejection in Early Adulthood?**

Using a whole-brain corrected (z = 2.3, p = .05) covariate analysis, we examined the association between teens’ empathic support provision to close peers across adolescence and neural responses to exclusion relative to inclusion in the Cyberball task. Clusters were defined based on structural probability maps in FSL and EBRAINS and by the continuity of functional activation. Higher teen empathic support provision was associated with reduced activation in a large cluster encompassing the subgenual cingulate area (x = −4, y = 10, z = −16; x = −8, y = 8, z = −16; x = 10, y = 8, z = −14), as well as left accumbens (x = −8, y = 10, z = −8), left caudate (x = −6, y = 14, z = 2), and left lateral ventricle (x = −2, y = 10, z = 4; Table S1; Figure S1). Multiple nearby peaks within this cluster indicate spatially consistent effects across ventral affective and subcortical regions. These results suggest that greater empathic support provision during adolescence is associated with attenuated neural responses to social exclusion in regions implicated in affective salience, reward, and social monitoring.

After adjusting for gender and baseline family income, higher adolescent empathic support provision was associated with reduced activation in the subgenual cingulate area (x = −10, y = 8, z = −18; p < .001; Figure S2, Table S2). Additional peaks of reduced activation were observed in striatal and subcortical regions, including the left (x = -8, y = 8, z = -10) and right nucleus accumbens (x = 8, y = 12, z = −8). Patterns of findings remained largely similar compared to main analyses where all timepoints were included.

**Figure S1.** The left accumbens and subgenual cingulate area (x = -8, y = 10, z = -8). Participants who provided greater empathic support to friends in adolescence showed reduced activity in this cluster when they were excluded, compared to when they were included in ball-tossing game, before adjusting for gender and baseline family income.

**
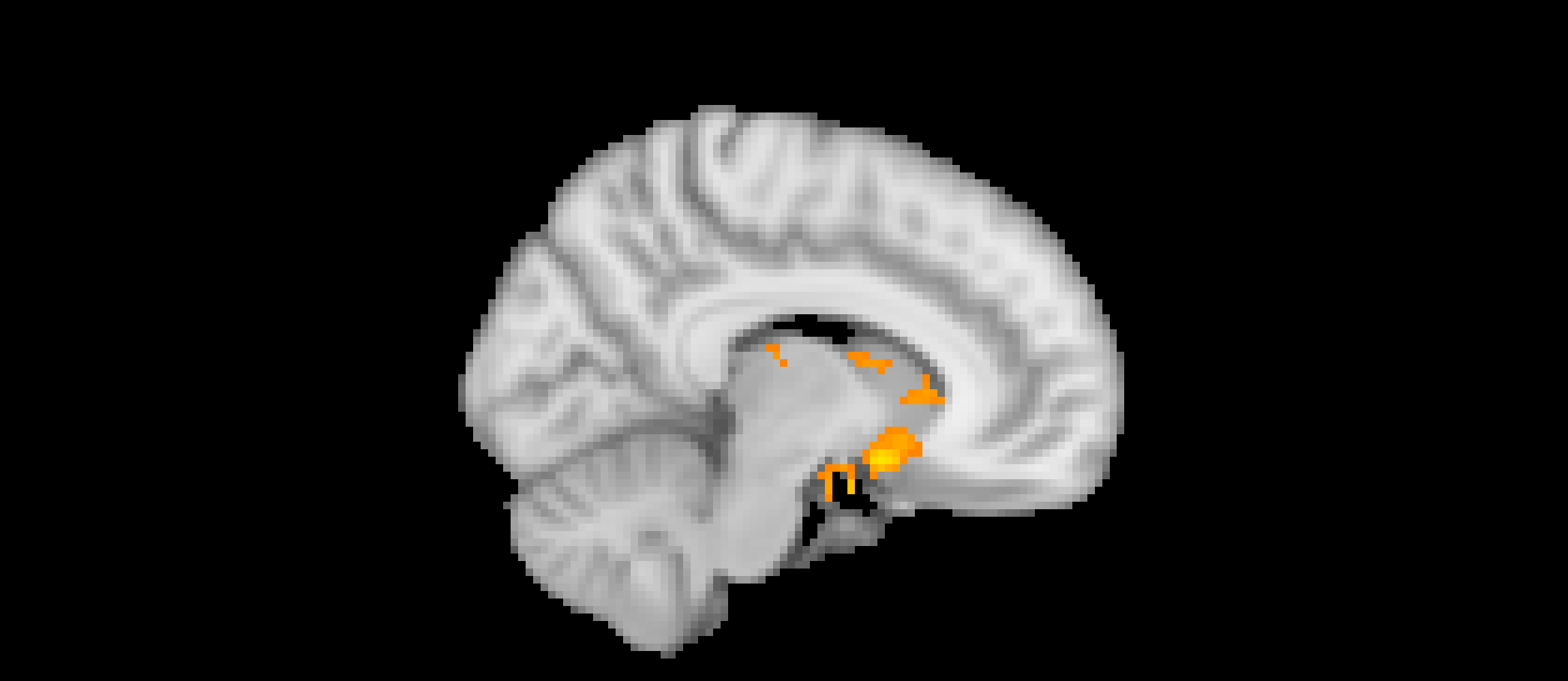
**

**Table S1.** Significant clusters of activity for the exclusion > inclusion contrast with teens’ empathic support provision index score before adjusting for gender and family income.

MNI coordinates

Structural location Cluster size in voxels Z-max X Y Z

Exclusion > Inclusion Cluster 2055

Left Accumbens 4.45 -8 10 -8

Subgenual Cingulate Area 4.42 -4 10 -16

Subgenual Cingulate Area 4.39 -8 8 -16

Left Caudate 4.18 -6 14 2

Left Lateral Ventricle 4.13 -2 10 4

Subgenual Cingulate Area 4.03 10 8 -14

*Note:* X, Y, and Z represent coordinates in relation to an origin, usually the anterior commissure, which specifies the coordinates of X = 0, Y = 0, and Z = 0. The x-axis goes towards the right side of the brain, the y-axis goes towards the front of the brain, and the z-axis goes towards the top of the brain. The orientation indicates which direction relative to the origin is positive or negative.

**Figure S2.** The subgenual cingulate area (x = -10, y = 8, z = -18). Participants who provided greater empathic support to friends in adolescence showed reduced sACC activity when they were excluded, compared to when they were included in ball-tossing game, after adjusting for gender and baseline family income.

**
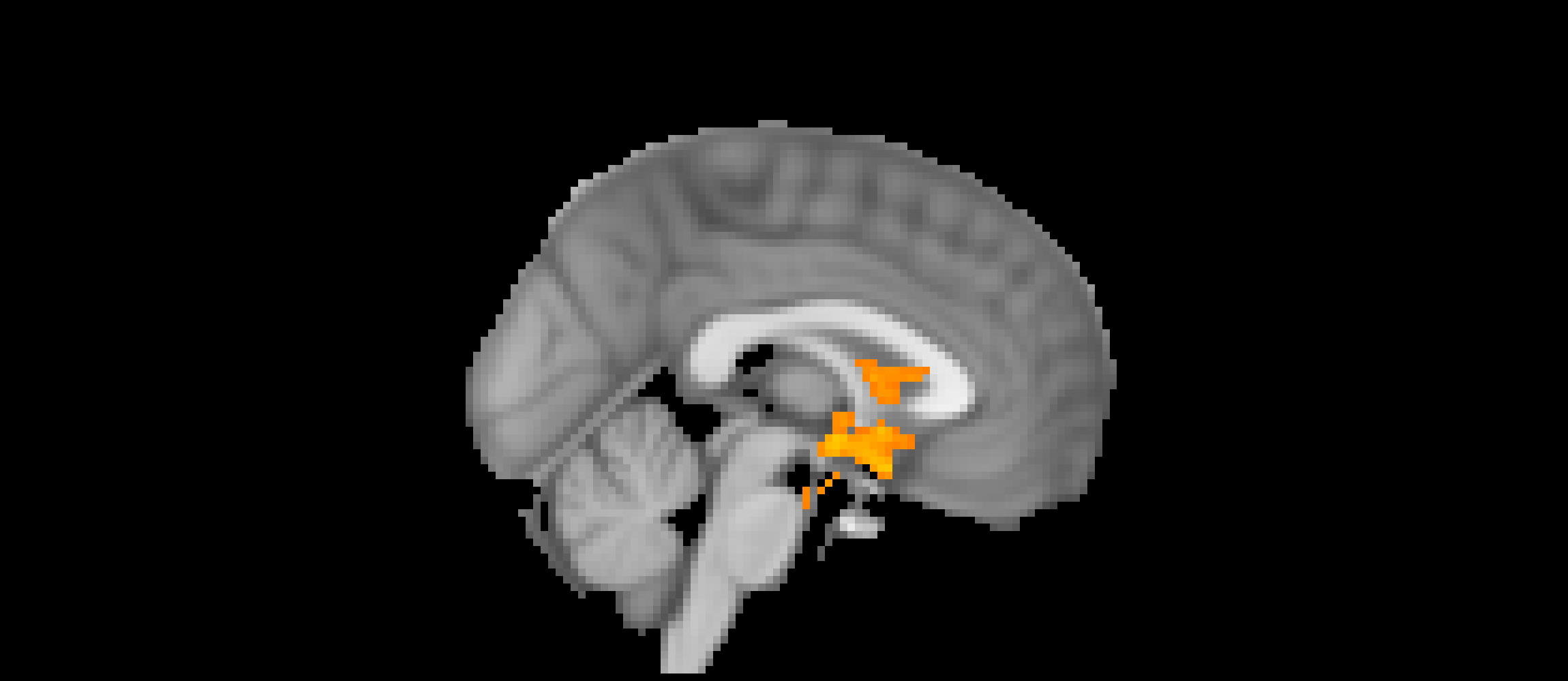
**

**Table S2.** Significant clusters of activity for the exclusion > inclusion contrast with teens’ empathic support provision index score after adjusting for gender and family income.

MNI coordinates

Structural location Cluster size in voxels Z-max X Y Z

Exclusion > Inclusion Cluster 1639

Subgenual Cingulate Area 4.71 -10 8 -18

Left Accumbens 4.42 -8 8 -10

Right Accumbens 4.33 8 12 -8

Right Accumbens 4.24 6 6 -8

Right Accumbens 4.07 10 8 -14

Left Caudate 3.84 -6 14 2

*Note:* X, Y, and Z represent coordinates in relation to an origin, usually the anterior commissure, which specifies the coordinates of X = 0, Y = 0, and Z = 0. The x-axis goes towards the right side of the brain, the y-axis goes towards the front of the brain, and the z-axis goes towards the top of the brain. The orientation indicates which direction relative to the origin is positive or negative.
